# Reducing therapeutic misconception: A randomized intervention trial in hypothetical clinical trials

**DOI:** 10.1371/journal.pone.0184224

**Published:** 2017-09-20

**Authors:** Paul P. Christopher, Paul S. Appelbaum, Debbie Truong, Karen Albert, Louise Maranda, Charles Lidz

**Affiliations:** 1 Department of Psychiatry & Human Behavior, Alpert Medical School, Brown University, Providence, RI, United States of America; 2 Division of Law, Ethics, and Psychiatry, Department of Psychiatry, Columbia University College of Physicians and Surgeons, New York, NY, United States of America; 3 Department of Psychiatry, University of Massachusetts Medical School, Worcester, MA, United States of America; 4 Department of Quantitative Health Sciences, University of Massachusetts Medical School, Worcester, MA, United States of America; University of Liverpool, UNITED KINGDOM

## Abstract

**Background:**

Participants in clinical trials frequently fail to appreciate key differences between research and clinical care. This phenomenon, known as therapeutic misconception, undermines informed consent to clinical research, but to date there have been no effective interventions to reduce it and concerns have been expressed that to do so might impede recruitment. We determined whether a scientific reframing intervention reduces therapeutic misconception without significantly reducing willingness to participate in hypothetical clinical trials.

**Methods:**

This prospective randomized trial was conducted from 2015 to 2016 to test the efficacy of an informed consent intervention based on scientific reframing compared to a traditional informed consent procedure (control) in reducing therapeutic misconception among patients considering enrollment in hypothetical clinical trials modeled on real-world studies for one of five disease categories. Patients with diabetes mellitus, hypertension, coronary artery disease, head/neck cancer, breast cancer, and major depression were recruited from medical clinics and a clinical research volunteer database. The primary outcomes were therapeutic misconception, as measured by a validated, ten-item Therapeutic Misconception Scale (range = 10–50), and willingness to participate in the clinical trial.

**Results:**

154 participants completed the study (age range, 23–87 years; 92.3% white, 56.5% female); 74 (48.1%) had been randomized to receive the experimental intervention. Therapeutic misconception was significantly lower (p = 0.004) in the scientific reframing group (26.4, 95% CI [23.7 to 29.1] compared to the control group (30.9, 95% CI [28.4 to 33.5], and remained so after controlling for education (p = 0.017). Willingness to participate in the hypothetical trial was not significantly different (p = 0.603) between intervention (52.1%, 95% CI [40.2% to 62.4%]) and control (56.3%, 95% CI [45.3% to 66.6%] groups.

**Conclusions:**

An enhanced educational intervention augmenting traditional informed consent led to a meaningful reduction in therapeutic misconception without a statistically significant change in willingness to enroll in hypothetical clinical trials. Additional study of this intervention is required in real-world clinical trials.

## Introduction

Clinical trials ask one or more basic scientific questions: Is a particular intervention safer, more tolerable, or more effective than other approaches to a given health condition? To answer this question, a protocol often involves randomization to one or more intervention arms, blinding of researchers and participants, constraints on dosing, limiting adjunctive treatments, and additional testing (e.g., biopsies, blood draws, imaging) to determine the consequences of an intervention. Such procedures depart dramatically from ordinary medical practice. Indeed, their use in routine medical care might be unethical because they limit the tailoring of treatment to patients’ individual needs and risk exposing patients to unnecessary harms.

Yet participants across a wide range of clinical research settings and trial designs often do not appreciate adequately how these indispensable features of trial research differ from the care they would otherwise receive outside of a study [1–4]. When clinical research participants fail to grasp key differences between participating in a clinical trial and receiving ordinary clinical care, they are said to manifest a therapeutic misconception. Previous research has suggested that therapeutic misconception can manifest in three ways: 1) an incorrect belief that treatment will be individualized to address a participant’s own needs, 2) the failure to realize that advancing scientific knowledge (as opposed to benefitting individual participants) is the primary purpose of a clinical trial [5], or 3) an unrealistic expectation of personal benefit from participation based on a misunderstanding of research methods [6].

Regardless of which components of therapeutic misconception have been examined, most studies have found that it is a common problem [2,5,6], and one that poses a challenge to the validity of informed consent [7,8]. When participants do not understand a trial’s purpose or how aspects of its methodology differ from usual medical care, their decision to join a research study necessarily rests on inadequate information. Ignoring these misconceptions devalues participants’ autonomy and dignity [8]. To date, no studies have examined ways to reduce therapeutic misconception among research participants. This may partly reflect a concern that participants would be unwilling to join studies if therapeutic misconceptions are dispelled [9,10].

As described elsewhere [11], therapeutic misconception arises in part from individuals’ tendency to view trial participation in a personal clinical frame, that is, with regard to their individual illnesses and treatment needs. The current study tests whether augmentation of traditional informed consent with an educational intervention designed to help participants reframe the elements of a clinical trial as a test of a scientific hypothesis is effective in reducing therapeutic misconception without decreasing willingness to participate in a clinical trial.

## Methods

### Study design and participants

The University of Massachusetts Medical School (UMMS) Institutional Review Board (IRB) approved a waiver of written informed consent for this minimal risk study; participants provided verbal consent after viewing a fact sheet that specified that the study involved: consideration of a hypothetical trial, viewing one of two informed consent presentations, and completion of a survey. Participants were compensated $20 and provided parking vouchers as needed.

A randomized trial was conducted to evaluate an enhanced disclosure based on scientific reframing of the methods of clinical trials for participants from five broad disease groups (cardiac disorders, cancer, diabetes, hypertension, and depression). Eligible individuals were all English-speaking adults (over 18) currently receiving treatment for the one of the five disease groups, who were not currently in and who clinicians did not expect would be asked to participate in an actual clinical trial, not diagnosed with a psychotic disorder, and able to provide informed consent. Individuals were excluded if they did not live within convenient traveling distance to participate, or if the research group was unable to obtain their contact information.

Recruitment occurred from May 2015 through October 2016, in the cardiology, oncology, psychiatry, and family medicine clinics at UMass Memorial Health Care (UMMHC) in the United States, with procedures varying somewhat across clinics. All clinics except cardiology had flyers placed in clinic waiting areas. In addition, clinicians in each clinic were asked to refer eligible patients to the study. Because of the number of on-going clinical trials, cardiology patients were pre-screened by clinical staff for eligibility before recruitment. Similarly, depression patients were pre-screened by clinicians for clinical appropriateness. Clinically referred patients who met eligibility checks and did not contact us directly received an introductory letter and a subsequent telephone call. To augment recruitment, hypertension and diabetes patients were directly approached in clinic waiting areas. Lastly, some participants were recruited from a UMMS research volunteer list.

Study staff screened potential participants to see if they were eligible for the study, i.e., receiving treatment for one of the five conditions and within convenient distance from the medical center—by reviewing their medical records. Research staff also confirmed diagnoses, treatment, and current non-participation in clinical trials with potential participants.

Participants were invited to learn about a hypothetical trial specific to their disease group. They were randomized (1:1) by a computerized random number generator to one of two arms (stratified by disease group): a control arm that was intended to mimic a standard consent procedure, and an experimental arm that provided a scientific reframing disclosure (described below) followed by the standard consent procedure. Participants were not made aware of the randomization process or that half of the participants would be receiving the reframing intervention. Hence, they had no way of identifying the disclosure they received as either experimental or control.

### Procedures

All participants viewed an informed consent disclosure for a hypothetical clinical trial targeting the disorder for which they were receiving treatment. Disclosures were developed in consultation with researcher-clinicians who had expertise in conducting trials for one of the five disease groups. The trials and corresponding informed consent disclosures were designed to reflect current, real-world, randomized controlled trials as listed on clinicaltrials.gov or as identified by the researcher-clinicians: 1) for cardiovascular disease, a trial comparing an experimental anti-inflammatory medication targeting atherosclerosis versus statin therapy (control) following intracoronary stent placement; 2) for cancer, either a) a trial comparing two experimental medications, each given in addition to a standard medication for locally recurrent or metastatic breast cancer, or b) a trial comparing radiation, cisplatin, and an experimental drug against radiation, cisplatin and a standard medication for head/neck cancer; 3) for major depression, a trial comparing an experimental intravenous medication versus a ketamine-like infusion for treatment-resistant major depression; 4) for diabetes mellitus, a trial comparing an experimental, ultra-rapid-acting inhaled insulin versus an approved inhaled insulin, 5) for hypertension, a trial comparing an approved versus an experimental direct renin inhibitor. All disclosures were embodied in a slide set with professional narration, which participants viewed on a tablet computer.

Prior to viewing the informed consent disclosure, the experimental group also viewed a scientific reframing intervention. Scientific reframing focused on providing education regarding the rationale behind clinical trials and the specific differences between clinical trial research and clinical care. This included five content areas: 1) That the purpose of the research is to assess whether the experimental intervention is more or less effective than the standard (control) treatment; that the reason the researchers are interested in doing this study is that they genuinely do not know whether the experimental treatment is better than the current standard of care; and that if they knew it was better there would be no need to do the study; 2) A description of randomization, including both the logic behind randomization (i.e., minimizing the risk of selection bias in assignment to the different arms of the study) and the inability of the researcher to affect assignment; 3) Limitations on dosage and adjunctive medications and why such limitations are important to the validity of the study; 4) The blinding of the subject and the physician as to which medication the subject is getting and how that will protect the study design from expectation bias; 5) That all of the above are done only to improve the scientific design and thus assure that the results of the study are valid and not to improve the care of the people in the study. In order to promote comprehension, particularly among individuals with lower literacy and/or visual impairments, the scientific reframing intervention was administered in a computerized slideshow, with professionally narrated slides containing text and animations to retain subjects’ interest and attention. The slides auto-advanced and took 12 minutes to view.

Both the informed consent disclosures and the scientific reframing intervention used in this study are publicly accessible at https://www.youtube.com/UMassSPARC/.

### Outcomes

Upon completion of the informed consent and (for the experimental group) reframing procedures, participants were provided a link to a RedCap survey. Each section of the survey had to be completed before moving to the next; once submitted, answers could not be reviewed or changed. All participants were given the option of having the survey items read to them in an interview format. The survey items were primarily self-administered, except for nine individuals who requested that items and response options be read aloud to them. Five of these participants reported a visual, hearing, or reading impairment. To assess willingness to participate in the hypothetical trial, participants were asked: “If you had to decide right now, would you decide to participate or not?” Therapeutic misconception was assessed using a ten-item measure previously developed and validated among 220 clinical trial participants (range 10–50) [12]. Participant demographic and background information were also collected.

### Statistical analysis

SPSS statistical software (version 23) was used for all analyses. To detect a minimal difference of 5 units in the therapeutic misconception score (which, based on the distribution of scores from the validation study of the measure [12], corresponds to a meaningful difference in therapeutic misconception), with type I error of 5% (2-sided) and 80% power, a target sample size was set at 154 participants, large enough to detect an effect size of 0.4025. Two-tailed t tests were used to compare unadjusted mean therapeutic misconception scores by intervention group, and a chi-square test was used to compare the percentage of participants willing to participate in the hypothetical trial. Factorial two-way ANOVAs were used to determine main and interaction effects of group assignment and select baseline characteristics on therapeutic misconception.

## Results

**Fig 1:**
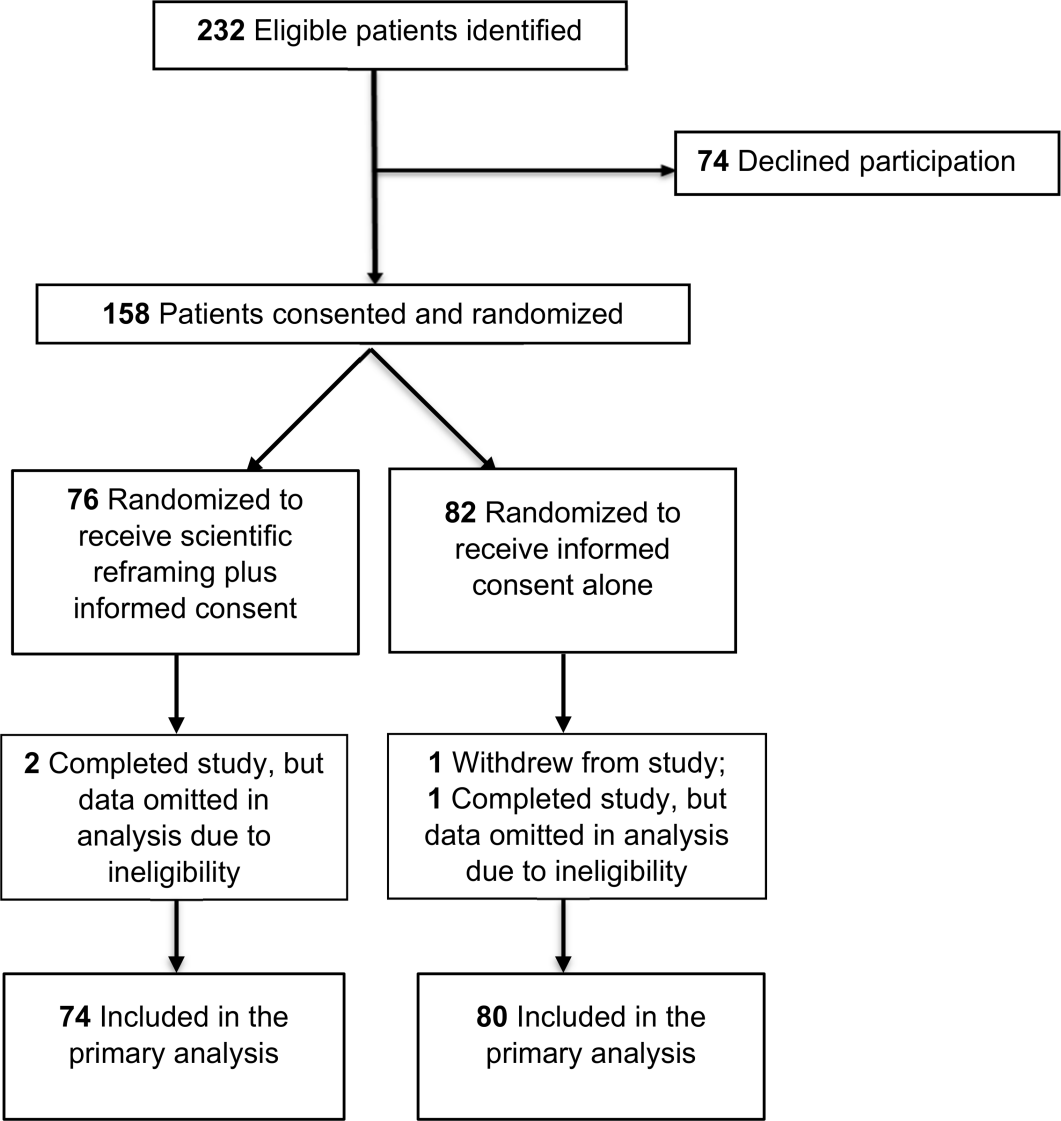
Flow diagram of a randomized trial comparing scientific reframing plus informed consent vs informed consent alone on therapeutic misconception in adults considering hypothetical clinical trial enrollment in 1 of 5 medical domains.

Between May 1, 2015 through October 31, 2016, two hundred thirty-two eligible patients were identified through all referral mechanisms combined (Fig 1). Of these, a total of 158 individuals (68%) consented to participate in the study: 82 were randomized into the control condition and 76 into the scientific reframing condition. After randomization, 1 participant in the control group quit the study. Two participants in the reframing group and 1 in the control group were omitted from the analysis due to a subsequent determination of ineligibility (i.e., a participant lacked fluency in English, another presented with cognitive disabilities, and a third later revealed that they were no longer receiving treatment for one of the index disorders). In all, 80 individuals in the control arm and 74 in the scientific reframing arm completed the study.

Participants’ mean age was approximately 55.9 years (Table 1). A majority were non-Latino (94.8%), white (90.3%), and female (56.5%); 37.0% had completed a college degree or higher; and 72.4% reported that they had not been employed in healthcare, higher education, or research.

**Table 1:** Participant characteristics, overall and by intervention group.

Characteristic	Total No. of Participants (n = 154)	Control (n = 80)	Scientific Reframing (n = 74)
	**N(%)**		
**Gender (male)**	67(43.5)	36(45.0)	31(41.9)
**Hispanic/Latino Ethnicity**	8(5.2)	3(3.8)	5(6.8)
**Race**			
White	139(90.3)	71(88.8)	68(91.9)
Black	7(4.5)	5(6.3)	2(2.7)
Asian	2(1.3)	1(1.3)	1(1.4)
More than one race	6(3.9)	3(3.8)	3(4.1)
**Educational Level**			
High school degree or less	43(27.9)	25(31.3)	18(24.3)
Some college or trade school	54(35.1)	30(37.5)	24(32.4)
College degree or more	57(37.0)	25(31.3)	32(43.2)
**Employment**^a^			
Health care, higher education, and research	42(27.6)	24(30.0)	19(26.0)
Other	110(72.4)	56(70.0)	54(74.0)
**Disease Group**			
Cardiology	30(19.5)	16(20.0)	14(18.9)
Oncology	30(19.5)	14(17.5)	16(21.6)
Depression	35(22.7)	19(23.8)	16(21.6)
Diabetes	33(21.4)	17(21.3)	16(21.6)
Hypertension	26(16.9)	14(17.5)	12(16.2)
**Age, years**^b^	**M(SD)**		
	55.9(12.1)	55.4(11.2)	56.5(13.1)

^a^ Employment for one participant in the scientific reframing group was not reported.

^b^ For participants for whom exact age was unknown (n = 9), the median value from the specified age range was used.

### Therapeutic misconception

Therapeutic misconception scores were significantly lower in the scientific reframing group (24.9, 95% CI [21.8 to 28.0]) compared to the control group (30.9, 95% CI [28.3 to 33.5]), with a mean difference of 6 points, 95% CI [1.97 to 10.04], (p = 0.004) (Table 2).

**Table 2:** Therapeutic misconception and willingness to participate, by intervention group.

	Control (n = 80)	Scientific Reframing (n = 74)	p value
	Mean score [95% CI]		
**Therapeutic Misconception**	30.9 [28.3 to 33.5]	24.9 [21.8 to 28.0]	0.004
	% [95% CI]		
**Willingness to Participate**	56.3 [45.3 to 66.6]	52.4 [40.2 to 62.4]	0.603

There was no significant main effect of disease group on therapeutic misconception scores, F(_4,144_) = 0.70, p = 0.594, and no significant interaction between disease group and intervention, F(_4,144_) = 0.45, p = 0.771.

**Fig 2:**
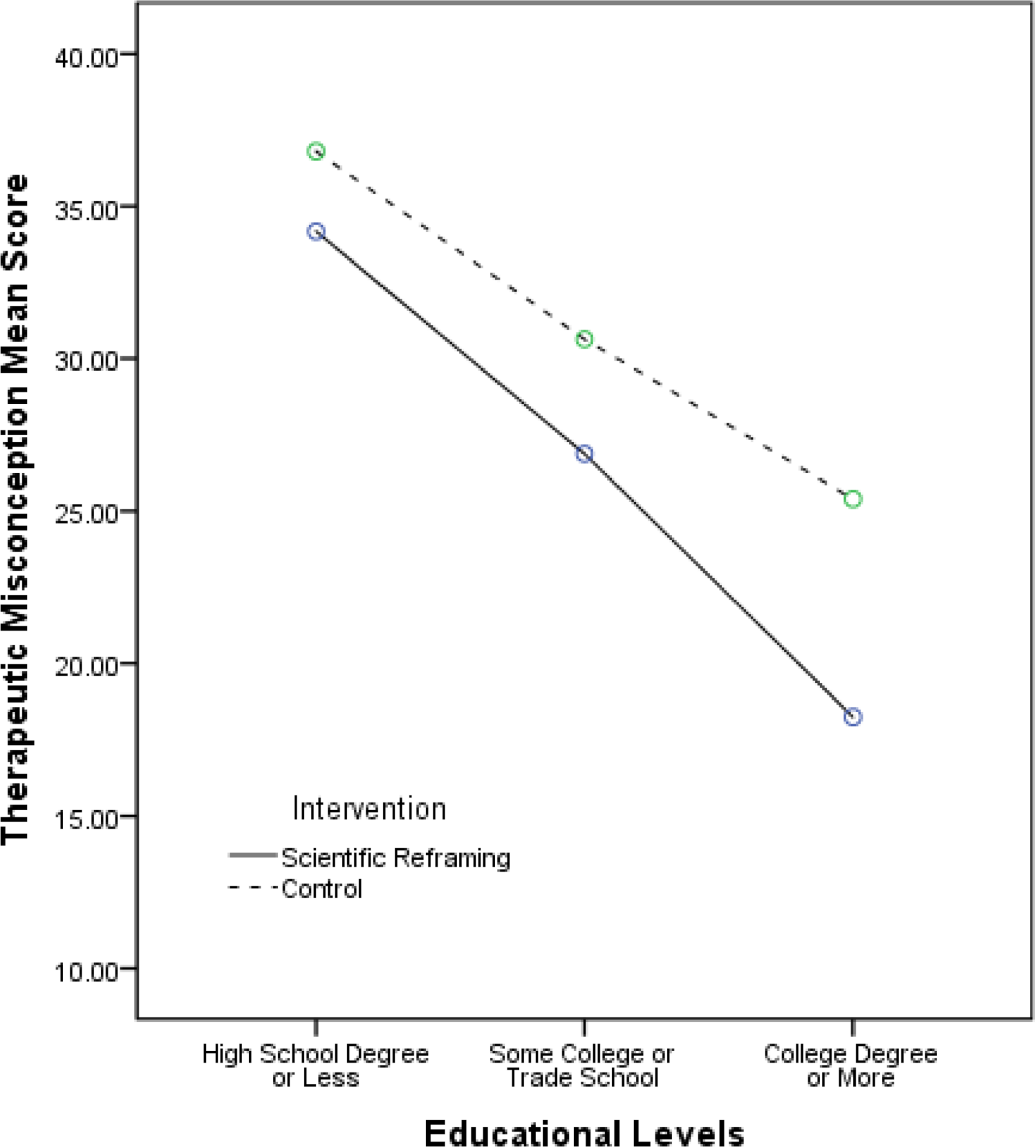
Group differences in therapeutic misconception scores by educational level.

Given previous data suggesting an inverse relationship between level of education and therapeutic misconception, we examined the effect of intervention and educational level concurrently and found that there was a significant main effect of educational level, F(2,148) = 17.2, p<0.001 (i.e., people with more education manifested lower levels of therapeutic misconception); the 95% CI around the b interaction coefficient of -7.15 was [-13.19 to -1.11]. The interaction between educational level and intervention was non-significant, F(_2,148_) = 0.54, p = 0.586; the 95% CI around the b coefficient of 3.39 was [-5.26 to 12.05]. See Fig 2.

### Willingness to participate

Expressed willingness to participate in the hypothetical clinical trial between scientific reframing (n = 38, 52.4%, 95% CI [40.2 to 62.4]) and control groups (n = 45, 56.3%, 95% CI [45.3 to 66.6]) did not differ to a significant degree (p = 0.603); see Table 2. Irrespective of intervention group, therapeutic misconception did not differ significantly (p = 0.91) between those who expressed willingness to participate (27.9, 95% CI [25.1 to 30.9]) and those who did not (27.8, 95% CI [24.6 to 30.8]).

## Discussion

Therapeutic misconception is an important and prevalent ethical problem in consent to clinical trials [1,7]. Poor appreciation of the core differences between clinical research and clinical care jeopardizes the validity of informed consent to trial participation. In this randomized trial, scientific reframing through an educational intervention was associated with a significant reduction in therapeutic misconception. The decrease in therapeutic misconception scores, a mean difference of six points, corresponds to a change from incorrect to correct answers on two or three items of this ten-item scale, or a reduction in strength of incorrect responses across a larger number of items. Consistent with past research [1–3], therapeutic misconception was higher among those with less educational attainment, although participants in the intervention group showed evidence of reduced therapeutic misconception across all education levels. To our knowledge, this is the first study to demonstrate the efficacy of an intervention specifically designed to reduce therapeutic misconception among patients similar to those who would be recruited for clinical trials. An appropriate next step is to test this intervention on participants in real clinical trials; based on the findings from this study, we would hypothesize that participants in actual trials will respond similarly to those in this study.

Despite previous concerns that efforts to dispel therapeutic misconception might deter individuals from enrolling in clinical trials, this study found preliminary evidence that willingness to participate did not differ between those in the intervention and control groups to a statistically significant degree. This finding and the observed interaction between education and the study intervention merit further examination as the present study was not powered to detect predetermined differences on these variables.

These findings have important implications for the current approach to conducting informed consent for clinical trials. Consent discussions customarily focus on the specific features of a trial, including the study’s purpose and procedures, and the potential risks and benefits of participation. Although this approach appropriately highlights the unique characteristics of the experimental intervention being tested, research suggests that when potential participants hear this information they tend to incorporate it into their existing cognitive framework based on their own illnesses and personal needs [11]. In other words, many view participation in a clinical trial as an extension of their treatment without necessarily grasping the broader, fundamental differences between clinical research and ordinary clinical care. The educational intervention tested in this study was designed to shift participants’ framework to help them appreciate how such differences undergird the validity of the scientific data that clinical trials seek to provide and, therefore, how clinical trials differ from accepted medical treatment. Much of the content included in the intervention has been characterized as essential for optimizing informed consent under a shared decision-making framework [13–15].

The design of the reframing intervention has additional strengths. First, the information that is covered, which clearly goes beyond what is disclosed in a typical informed consent discussion, is relevant to a variety of clinical trial designs and thus can be applied with little or no modification to many other trial settings. Moreover, by administering the intervention prior to the typical informed consent discussion, prospective participants are given a context in which to incorporate subsequent information about the trial to which they are being recruited. In this regard, the intervention may be preventing, as opposed to merely correcting, therapeutic misconception. Third, in its current form, the intervention is 12 minutes long, which amounts to a fraction of the time required for an informed consent discussion in a typical clinical trial.

Clinical trials often experience difficulty meeting target recruitment goals [16], and some authors have speculated that redressing therapeutic misconception might further slow clinical trial recruitment [9,10]. In the present study, those who received the intervention were not less interested to a statistically significant degree in participating than those in the control group, nor did therapeutic misconception scores differ between those inclined to participate and those who were not. Although we know of no other work examining the impact of an intervention to address therapeutic misconception on enrollment decisions, a recent review of other audio-visual informed consent interventions found they do not undermine clinical trial recruitment and that their effect on understanding is extremely low [17]. As noted, however, our finding requires replication with attention paid to whether reducing therapeutic misconception affects both recruitment and retention in large clinical trials. Nevertheless, if this finding is confirmed, it should not be surprising. People have a variety of reasons for enrolling in clinical trials, including altruism, and many individuals without therapeutic misconception may have realistic estimates of receiving some degree of personal benefit. Put another way, redressing therapeutic misconception does not require pessimism on the part of potential participants about what a clinical trial may provide. Indeed, clinical trials can grant patients access to resources that may be otherwise unavailable, including promising investigational agents, expert medical evaluation, and close monitoring of their disorders. Thus, at present, the fear that confronting therapeutic misconception will thwart the advance of medical science seems misplaced.

A number of study limitations should be noted. First, in response to the concern that reducing therapeutic misconception might jeopardize enrollment, we intentionally chose to test the intervention initially in hypothetical clinical trials; this approach limits the generalizability of study findings. An appropriate next step is to test the intervention in actual clinical trials. Second, despite constructing hypothetical trials that reflect current clinical trial research in five different disease categories, there are clearly a multitude of diseases that we did not include in this study as well as other clinical trial designs, including pragmatic and placebo-controlled trials. These warrant testing in the future. Third, this study was powered to detect a difference in therapeutic misconception score but not willingness to participate, or interactions between the outcomes and non-intervention variables. Finally, the participants in this study were disproportionately white, female and well-educated; the efficacy of scientific reframing in other populations remains to be determined.

## Conclusions

In the context of hypothetical clinical trials in five medical domains, this study found that therapeutic misconception can be substantially reduced through a non-burdensome scientific reframing intervention that augments existing informed consent practices, without jeopardizing enrollment to a statistically significant degree. Given the ethical importance of therapeutic misconception, further testing of this intervention in real-world clinical trials is warranted.
